# The Effect of Immune Checkpoint Inhibitor Therapy on Pre-Existing Gastroparesis and New Onset of Symptoms of Delayed Gastric Emptying

**DOI:** 10.3390/cancers16152658

**Published:** 2024-07-26

**Authors:** Andres C. Urias Rivera, Antonio Pizuorno Machado, Malek Shatila, George Triadafilopoulos, Jennifer L. McQuade, Mehmet Altan, Dan Zhao, Yinghong Wang, Mehnaz A. Shafi

**Affiliations:** 1 Department of Internal Medicine, Baylor College of Medicine, Houston, TX 77030, USA; andres.uriasrivera@bcm.edu; 2 Department of Internal Medicine, The University of Texas Health Science Center, Houston, TX 77030, USA; 3 Department of Gastroenterology, Hepatology and Nutrition, The University of Texas MD Anderson Cancer Center, Houston, TX 77030, USAywang59@mdanderson.org (Y.W.); 4 Department of Melanoma Medical Oncology, The University of Texas MD Anderson Cancer Center, Houston, TX 77030, USA; 5 Department of Thoracic/Head & Neck Medical Oncology, The University of Texas MD Anderson Cancer Center, Houston, TX 77030, USA; 6 Department of Gastrointestinal Medical Oncology, The University of Texas MD Anderson Cancer Center, Houston, TX 77030, USA

**Keywords:** delayed gastric emptying, gastroparesis, immune checkpoint inhibitors, immune-related adverse events

## Abstract

**Simple Summary:**

Immune checkpoint inhibitors (ICIs) offer improved outcomes for patients with various malignancies but they have also been associated with immune-related adverse events. Gastroparesis, a gastrointestinal (GI) motility disorder characterized by delayed gastric emptying (GE) and upper GI symptoms, has been linked to ICIs through histological studies indicating myenteric plexopathy. The aim of our retrospective study was to evaluate the prevalence, clinical course, and treatment for both patients with pre-existing gastroparesis and those with new symptoms of delayed GE after ICI. We found 39 patients with new delayed GE post-ICI (prevalence of 0.2%) and 37 with pre-existing gastroparesis, of which only 4 (11%) had a flare of symptoms post-ICI. We found that delayed GE post-ICI therapy is rare but with late onset and prolonged duration. The presence of alternative etiology of gastroparesis did not affect patients’ duration of delayed GE symptoms or overall survival.

**Abstract:**

Immune checkpoint inhibitors (ICIs) can cause myenteric plexopathy, which could result in delayed gastric emptying (GE) and possibly gastroparesis. We assessed the clinical outcomes of patients who had pre-existing gastroparesis or who developed symptoms of delayed GE following ICI therapy. We retrospectively identified adults with ICD-9 and ICD-10 codes for gastroparesis who received ICI therapy between 1 January 2020 and 31 December 2022 at a tertiary cancer center. Of 76 eligible patients, 37 had pre-existing gastroparesis; 39 (0.2% of the more than 18,000 screened) developed symptoms of delayed GE after ICI therapy, of which 27 (69%) patients had an alternative etiology for delayed GE. Four patients (11%) with pre-existing gastroparesis had a flare-up after ICI, and the median time to flare-up was 10.2 months (IQR, 0.7–28.6 months); for patients with new onset of suspected delayed GE after ICI, the median time to symptom onset was 12.8 months (IQR, 4.4–35.5 months). The clinical symptom duration of patients without an alternative etiology (74.5 days (IQR, 21.5–690 days)) and those with an alternative etiology (290 days (IQR, 147–387 days)) did not differ significantly (*p* = 1.00). Delayed GE after ICI therapy is a rare presentation but has a late onset and a prolonged symptom duration.

## 1. Introduction

Immune checkpoint inhibitors (ICIs) are monoclonal antibodies that block cytotoxic T lymphocyte-associated antigen 4 (CTLA-4), programmed cell death protein 1 (PD-1), or programmed cell death ligand 1 (PD-L1), which downregulate antitumor T-cell activity [1]. Owing to their off-target inflammatory effects in healthy cells, ICIs can cause immune-related adverse events (irAEs), most commonly gastrointestinal (GI) irAEs, in up to 65% of patients [2,3].

Gastroparesis, a motility disorder of delayed gastric emptying (GE) in the absence of mechanical obstruction, is associated with upper GI symptoms, including nausea, vomiting, early satiety, and upper abdominal pain. An idiopathic etiology, seen in one-third of patients with gastroparesis, is most common. The most common systemic disease associated with gastroparesis is diabetes [4]. Other etiologies include surgery (commonly gastric or thoracic surgery resulting in vagal nerve injury), infection, medications (commonly, opioids, GLP-1 agonists, and tricyclic antidepressants), neuromuscular diseases, infiltrative disorders, pyloric dysfunction, autoimmune/paraneoplastic processes, and oxidative stress/inflammation [4,5]. Upper GI motility is modulated by an interplay among the enteric nervous system, via the pacemaker myenteric interstitial cells of Cajal (ICCs), the autonomic nervous system, and nitrergic pathways that control pyloric motor activity and other GI smooth muscle cells [6,7]. The two most common gastric cellular defects in gastroparesis are the loss of ICC and neuronal nitric oxide synthase [8], which studies using animal models have shown may be related to the effects of immune injury [9].

Researchers have proposed several hypotheses explaining how ICIs can induce delayed GE. One hypothesis is that ICIs reduce the levels of anti-inflammatory M2 macrophages, thereby decreasing heme oxygenase-1 expression and ultimately increasing oxidative stress and inflammation, which in turn damages the ICCs. Low levels of M2 macrophages have been correlated with the loss of ICCs in both idiopathic and diabetic gastroparesis [4,10]. Case reports of GI motility disorder arising after ICI therapy, in which deep tissue biopsies of the colon showed either increased C4d immunoreactivity or dense peri-neural T-cell infiltrate surrounding neurons and disrupting nerve fibers within the myenteric plexus, support the hypothesis that ICIs can also increase cytotoxic T-cell and humoral (paraneoplastic) immunity, causing myenteric plexopathy [11,12]. Yet another hypothesis is that the shared expression of gangliosides, Hu, or other antigens or other shared major histocompatibility complex epitopes on neurons and tumor cells predisposes patients to immune toxicity upon the initiation of ICI therapy [12,13,14,15].

Studies of ICIs’ impact on gastric motility are limited, comprising just a few case series or reports of patients without pre-existing gastroparesis who received PD-1 or CTLA-4 inhibitors for melanoma, breast cancer, Merkel cell carcinoma, neuroendocrine tumors, or lung cancer [12,15,16]. The diagnosis of ICI-induced gastroparesis is difficult to make conclusively given the nonspecific symptoms and variable time of onset, which ranged from 2 to 38 months in a case series by Atieh et al. [16]. Diagnostic studies such as gastric scintigraphy, the wireless motility capsule test, and the stable isotope breath test are not routinely performed in clinical practice. In the case series by Atieh et al., only 19 of 1300 symptomatic patients screened had undergone gastric scintigraphy after ICI therapy, and of these 19 patients, only 3 had delayed GE [16]. Full-thickness biopsies to identify enteric nervous system pathology are rarely performed. These diagnostic challenges could lead to an under-reporting and false low prevalence of ICI-induced gastroparesis, as noted in a systematic review in which only 2 cases of isolated enteric neuropathy were identified from 428 cases of ICI-induced neurological irAEs spanning 649 studies [17]. Guidelines for the management of ICI-related gastroparesis are also minimal, as most guidelines focus on common ICI-related GI toxicities, such as colitis [18]. Thus, there is a critical need for further investigation of the prevalence and clinical outcomes of gastroparesis and delayed GE post-ICI therapy, both for patients with a new onset of symptoms and those with pre-existing gastroparesis.

## 2. Materials and Methods

### 2.1. Patients

This is a retrospective cohort analysis of cancer patients whose medical records included ICD-9 and ICD-10 codes for gastroparesis in the context of the receipt of ICI therapy between 1 January 2010 and 31 December 2022 at a tertiary cancer center. *Inclusion* criteria were as follows: (1) aged 18 years or older; (2) receipt of ICI therapy; (3) upper GI symptoms of nausea, vomiting, and/or upper abdominal pain; and (4) GI symptom onset starting after the initiation of ICI therapy and up to 1 year after its completion. We also analyzed patients who had a pre-existing diagnosis of gastroparesis (based on chart documentation or evaluation) before ICI initiation and evaluated their disease course. Gastric scintigraphy and endoscopy were not required for inclusion. *Exclusion* criteria were as follows: (1) no symptoms suspicious for gastroparesis; (2) no records of gastroparesis or suspected delayed GE; (3) a normal 4-h gastric scintigraphy; (4) the presence of mechanical obstruction; and (5) obstructive GI malignancy. We recorded other alternative etiologies for gastroparesis and analyzed their effect on clinical outcomes, and these included diabetes, opioid use, prior bariatric surgery, and localized GI malignancy. Data regarding demographics, diagnostic studies, treatments, and clinical outcomes were recorded and analyzed. 

### 2.2. Definitions

A flare-up of pre-existing gastroparesis was defined as an increase in dosage or frequency of medication to control delayed GE symptoms when previously unchanged for 3 months prior to the initiation of ICI. Clinical remission of delayed GE symptoms was defined as the discontinuation of prokinetic agents or no new refills of prokinetic medications for 3 months after the last visit in patients with minimal or resolved upper GI symptoms.

### 2.3. Diagnostic Study

Patients who underwent gastric scintigraphy at our institution were given a ^99m^Tc-labeled standardized meal, and then serial static images were taken throughout the 4 h study period. Gastric retention of more than 60% at 2 h or more than 10% at 4 h was considered delayed GE. An example of a gastric emptying study is included in Supplementary Figure S1.

### 2.4. Statistical Analysis

Categorical variables were described using frequencies and percentages. Continuous variables were described using medians and interquartile ranges (IQRs). The Fisher exact test was used to compare differences between categorical variables, and the Mann–Whitney U test was used to compare differences between continuous variables. Kaplan–Meier curves were used to analyze overall survival, which was defined as the time from the start of ICI therapy to the last follow-up or death from any cause. *P*-values less than 0.05 were considered to indicate statistical significance. All statistical analyses were performed with SPSS version 29.0.

## 3. Results

### 3.1. Patient Characteristics

**Figure 1:**
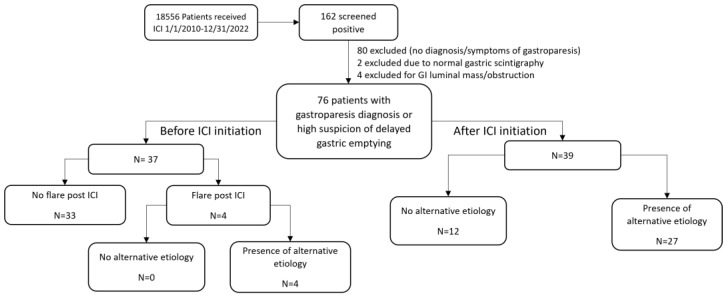
Patient selection flowchart.

Of the 18,556 patients who received ICI therapy during the study period, 76 had suspected delayed GE and met the study inclusion criteria (Figure 1). These patients’ characteristics are given in Table 1. Of these 76 patients, 37 had pre-existing gastroparesis before ICI initiation and 39 developed delayed GE symptoms after ICI initiation (post-ICI group). The median age of the pre-existing gastroparesis group (59.4 years (IQR, 45.0–65.7 years)) was significantly lower than that of the post-ICI group (64.5 years (IQR, 55.4–68.5 years); *p* = 0.034). The most frequent cancer type in the pre-existing gastroparesis group was GI cancer (35% (13/37)); in the post-ICI group, it was lung/head and neck cancer (31% (12/39)). In both groups, the most common ICIs patients received were PD-1 or PD-L1 inhibitors. Overall, the irAE rate of the pre-existing gastroparesis group (3% (1/37)) was significantly lower than that of the post-ICI group (33% (13/39); *p* = 0.001). The median follow-up durations after ICI therapy for the pre-existing gastroparesis and post-ICI groups were 1.7 and 2.1 years, respectively. Other parameters, such as all-cause mortality, did not differ significantly between the two groups. In the pre-existing gastroparesis group, four (11%) patients had a flare-up of symptoms requiring an increased prokinetic drug dosage.

The characteristics of the disease courses of the 37 patients who had pre-existing gastroparesis with (n = 33) or without (n = 4) a flare-up after ICI therapy and the 39 patients who had new onset of suspected delayed GE after ICI therapy are given in Table 2. In all three groups, nausea and vomiting were the predominant presenting symptoms, and metoclopramide was the medication most commonly given for presumed gastroparesis. Of the 39 patients who had suspected delayed GE after ICI therapy, 27 (69%) had an alternative etiology for delayed GE symptoms. The most common alternative etiology was opioid use 16 (41%), followed by diabetes 13 (37%). Only four patients (10%) with symptoms of delayed GE after ICI therapy underwent 4 h gastric scintigraphy. The median time from the initiation of ICI therapy to the onset of symptoms of delayed GE was 10.2 months (IQR, 0.7–28.6 months) for patients who had pre-existing gastroparesis with a flare-up after ICI and 12.8 months (IQR, 4.4–35.5 months) for those with new onset of suspected delayed GE after ICI therapy. Clinical remission was achieved in 14 (36%) patients who had a new onset of suspected delayed GE after ICI initiation and in 1 (25%) patient who had a flare-up of a pre-existing condition. Post-ICI patients had a median symptom duration of 108 days (IQR, 14.5–780 days). Among the 39 patients who had a new onset of suspected delayed GE, symptoms of delayed GE alone did not lead to the termination of ICI therapy; however, 12 patients (31%) stopped ICI therapy because of other irAEs. 

### 3.2. Subgtoup Analysis of Patients Stratified by the Presence of Alternative Etiologies for Upper GI Symptoms

The characteristics of the 39 patients with suspected new gastroparesis or delayed GE after ICI therapy stratified by the presence of alternative etiologies for upper GI symptoms are given in Table 3. Of the 27 patients with alternative etiologies, 3 (11%) underwent gastric scintigraphy, whereas 1 (8%) of the patients without alternative etiologies underwent gastric scintigraphy. The symptoms did not differ significantly between these two groups. The median duration of clinical symptoms did not differ significantly between the patients with no alternative etiology (290 days (IQR, 147–387 days)) and patients with an alternative etiology (74.5 days (IQR, 21.5–690.0 days); *p* = 1.00). Other variables, including gastroparesis medication, HbA1c level, and hospitalizations for gastroparesis-like symptoms, did not differ significantly between the two groups.

A comparison of disease courses between patients with suspected delayed GE after ICI therapy without an alternative etiology and patients with pre-existing gastroparesis without a flare-up after ICI therapy can be seen in Supplemental Table S1. The rates of nausea (100% (33/33)) and vomiting (94% (31/33)) for patients with pre-existing gastroparesis without a flare-up after ICI therapy were significantly higher than those for patients with suspected delayed GE after ICI therapy without an alternative etiology (75% (9/12); *p* = 0.016 and 58% (7/12); *p* = 0.010, respectively). There were no other differences in measured variables between the groups.

### 3.3. Survival

**Figure 2:**
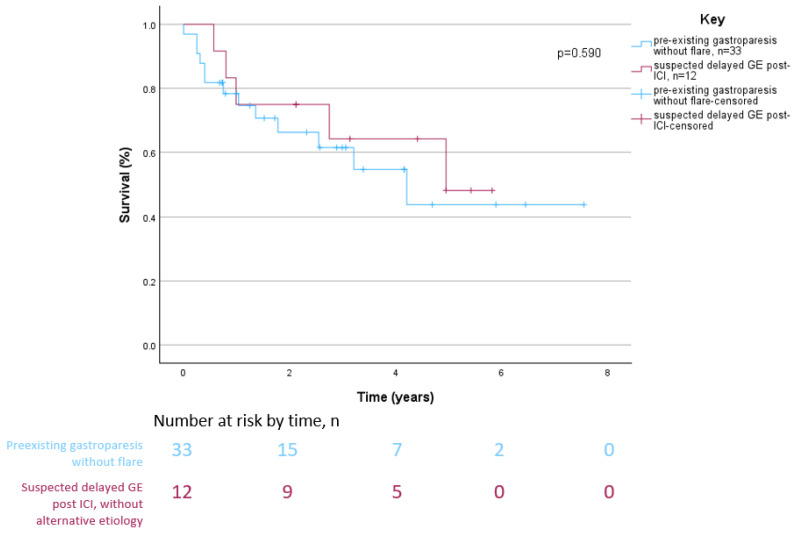
Overall survival of patients with pre-existing gastroparesis without flare-up vs. patients with delayed gastric emptying after immune checkpoint inhibitor therapy without an alternative etiology.

**Figure 3:**
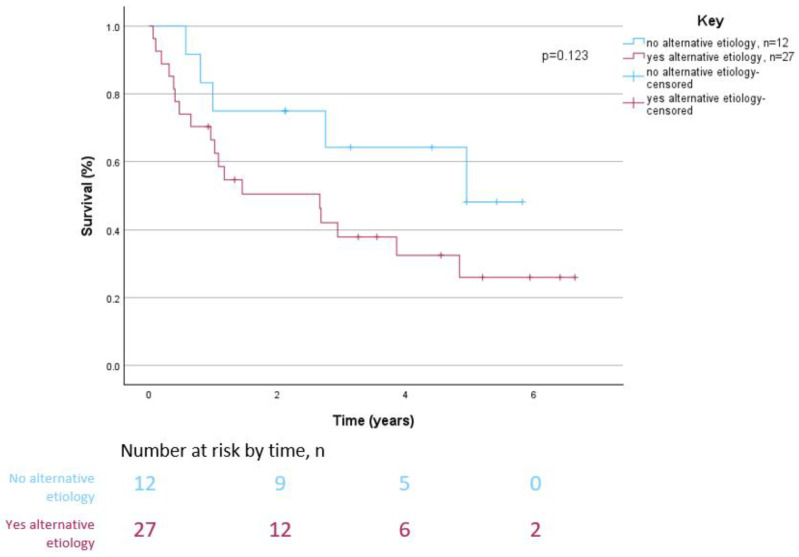
Overall survival of patients with suspected delayed gastric emptying after immune checkpoint inhibitor therapy by alternative etiology status.

Kaplan–Meier analyses showed that overall survival did not differ significantly between patients with suspected delayed GE without an alternative etiology after ICI therapy and patients with pre-existing gastroparesis without flare-up after ICI therapy (*p* = 0.590) (Figure 2). Similarly, overall survival did not differ significantly between patients without an alternative etiology and patients with an alternative etiology for delayed GE after ICI therapy (*p* = 0.123) (Figure 3).

## 4. Discussion

In the present study, only 39 (0.2%) of the more than 18,000 patients who received ICI therapy at our institution during the 13-year study period had a new onset of suspected delayed GE after ICI initiation. The onset of delayed GE post-ICI is variable but can be delayed with a median time to symptoms of over one year, with the duration of symptoms lasting a median of over 3 months. Flare-ups from pre-existing gastroparesis after ICI therapy are rare at around 11%. Sixty-nine percent of patients who had a new onset of suspected GE after ICI therapy had an alternative etiology for symptoms of delayed GE. The presence of an alternative etiology for delayed GE did not affect treatment response or overall survival. The overall response rate to standard therapy, mostly metoclopramide, was 36%; this finding is similar to those of a small randomized controlled trial enrolling 15 patients with idiopathic gastroparesis, in which metoclopramide improved symptoms in 42% of patients and improved GE in 36% [19].

Diagnosing gastroparesis in the cancer population is difficult, as many patients are on pain modulators that not only can affect gastroparesis diagnostic studies but also are associated with sustained symptom burden [20]. In addition, confounding factors, including other upper GI irAEs, coexisting neuropathy or diabetes, prior radiation, and even the malignancy itself, could contribute to gastroparesis [21,22]. In our study, patients with an alternative etiology for delayed GE had higher rates of nausea and vomiting but had a disease course that was similar overall to that of patients with no alternative etiology. However, among patients who had clinical remission, the median clinical symptom duration for those with no alternative etiology (290 days (IQR, 147–387 days)) was 4 times that for patients with an alternative etiology (74.5 days (IQR, 21.5–690)), but this difference was not statistically significant. This trend might indicate that ICI-associated delayed GE, in the absence of an alternative etiology, is more refractory to treatment than those with alternative etiologies for delayed GE. This would differ from previous reports, as idiopathic gastroparesis often requires fewer prokinetic agents and advanced interventions than gastroparesis associated with an alternative etiology (e.g., diabetes) [23]. Further studies with larger sample sizes are needed to help advance our understanding of the disease behavior.

One study found that the cumulative probabilities of irAE onset at 6, 12, and 24 months after ICI initiation were 42.8%, 51.0%, and 57.3%, respectively [24]. Most other studies corroborate these findings, with the onset of irAEs usually occurring within 3 months after ICI initiation. In a case series of patients with gastroparesis after ICI therapy, the time to symptom onset ranged from 2 to 38 months [16]. In our study, the median time to the onset of delayed GE after ICI therapy was 12.8 months (IQR, 4.4–35.5 months). The later onset of ICI-associated delayed GE may contribute to its underdiagnosis as clinical providers may not consider ICI-related irAEs in the differential diagnosis even 1 year after ICI therapy completion.

Patients with pre-existing autoimmune diseases have an increased risk of flare-ups after ICI therapy [25,26]. One single-center retrospective study found that 14.7% of patients with a pre-existing autoimmune disorder had a flare-up after ICI therapy [27]. Another study found that 41% of patients with inflammatory bowel disease had a GI adverse event after 2 months of ICI therapy initiation [28]. A recent single-cell multiomics analysis of human gastric muscle immune cells found a greater abundance of monocyte-like macrophage clusters and increased IL12-mediated JAK-STAT and EPH-ephrin signaling in idiopathic gastroparesis, further connecting immune dysregulation to the possible pathophysiology of gastroparesis [29]. Interestingly, we found that only four (11%) patients with pre-existing gastroparesis experienced a flare-up after ICI, with a median time to onset of 10.2 months (IQR, 0.7–28.6 months). Only one (25%) of these four patients had GI symptom remission, whereas 14 (36%) of the 39 patients with suspected delayed GE after ICI therapy had symptom remission. Patients with pre-existing autoimmune conditions who have flare-ups after ICI therapy frequently have suboptimal responses to medical treatment, and further studies to optimize these patients’ management and identify additional potential treatments are warranted.

The effective assessment and management of gastroparesis in relation to ICI requires a multifaceted approach. Increased use of gastric emptying studies to confirm delayed emptying and use of validated scores like the Gastroparesis Cardinal Symptom Index facilitate standardized monitoring and earlier intervention [30]. Diagnostic studies and interval symptom index scores are crucial for patients with pre-existing gastroparesis as they appear to be susceptible to flare-ups post-ICI therapy. Earlier diagnosis of pre-existing gastroparesis flare-up or new gastroparesis post-ICI can expedite a trial or enhanced treatment of gastroparesis, prior to ICI therapy, and if needed, the patient can be referred to a motility expert for neuromodulators, adjustment of opioids, or advanced endoscopic interventions [31]. There may be a role for steroid use for gastroparesis induced by ICI, because, as discussed previously, there are histological studies linking an immunological response to onset of gastroparesis post-ICI.

Our study had several limitations, including its retrospective nature and completion at a single center. In addition, the ICD codes we used for the initial data extraction may not have been accurately captured or entered into the database, and these potential errors, along with the lack of routine gastric scintigraphy, could have led us to underestimate the incidence of delayed GE. Some patients had alternative etiologies for gastroparesis with overlapping symptoms and co-existing conditions, and effectively separating these factors is highly complex and challenging.

## 5. Conclusions

In conclusion, our findings show that symptoms of delayed GE that are suggestive of gastroparesis after ICI therapy are rare but may have a late onset and prolonged duration. Patients frequently have alternative etiologies but these do not affect treatment response or overall survival. Flare-ups of pre-existing gastroparesis after ICI are rare. For patients on ICI therapy who have a new onset of upper GI symptoms, abnormal GI motility should be considered, and targeted diagnostic and medical management should be sought.

## Figures and Tables

**Table 1:** Patient characteristics.

Variable	Patients with Pre-Existing Gastroparesis, *n* = 37	Patients with New Onset of Suspected Delayed GE after ICI Therapy, *n* = 39	*p*
Median age at delayed GE onset (IQR), years	59.4 (45.0–65.7)	64.5 (55.4–68.5)	0.034
Male sex	19 (51)	26 (67)	0.243
Cancer type			0.003
Gastrointestinal	13 (35)	6 (15)	
Biliary/liver	3 (23)	1 (17)	
Esophagus	4 (31)	2 (33)	
Gastric/Duodenal	4 (31)	1 (17)	
Colon/Rectum/Other	2 (15)	2 (33)	
Lung/head and neck	8 (22)	12 (31)	
Pharynx/Tonsil	2 (25)	4 (33)	
Lung	6 (75)	8 (67)	
Melanoma	5 (14)	7 (18)	
Gynecological	5 (14)	1 (3)	
Hematological	5 (14)	1 (3)	
Genitourinary	1 (3)	10 (26)	
Endocrine	0 (0)	2 (5)	
ICI type			0.328
CTLA-4 inhibitor	5 (14)	4 (10)	
PD-1/PD-L1 inhibitor	28 (77)	29 (74)	
Combination of both	4 (11)	6 (14)	
Flare-up of pre-existing gastroparesis after ICI therapy	4 (11)	--	--
irAE after ICI therapy	1 (3)	13 (33)	0.001
Hepatitis	1 (100)	3 (23)	
Colitis	0 (0)	1 (8)	
Pancreatitis	0 (0)	2 (15)	
Hypophysitis	0 (0)	2 (15)	
Vasculitis	0 (0)	1 (8)	
Pneumonitis	0 (0)	4 (31)	
All-cause mortality	15 (41)	23 (59)	0.168
Median follow-up duration after ICI initiation (IQR), years	1.7 (0.7–3.8)	2.1 (0.8–4.6)	0.543

Note: All data are no. of patients (%) unless otherwise indicated. Abbreviations: Gastric emptying (GE), immune checkpoint inhibitor (ICI), interquartile range (IQR), cytotoxic T lymphocyte–associated antigen 4 (CTLA-4), programmed cell death protein 1 (PD-1), programmed cell death ligand 1 (PD-L1), immune-related adverse event (irAE).

**Table 2:** Characteristics of the disease course of patients with pre-existing gastroparesis or new onset of suspected delayed gastric emptying (GE) after immune checkpoint inhibitor (ICI) therapy.

Characteristic	Pre-Existing Gastroparesis without Flare-Up after ICI Therapy, *n* = 33	Pre-Existing Gastroparesis with Flare-Up after ICI Therapy, *n* = 4	New Onset of Suspected Delayed GE or Gastroparesis after ICI Therapy, *n* = 39
Alternative etiology for gastroparesis	26 (79)	4 (100)	27 (69)
Diabetes	9 (27)	1 (25)	13 (37)
Prior bariatric procedures	4 (12)	0 (0)	0 (0)
GI cancer	11 (33)	2 (50)	6 (15)
Medications			
Opioids	13 (39)	1 (25)	16 (41)
PPI	18 (54)	1 (25)	23 (59)
H2RA receptor antagonist	1 (3)	0 (0)	1 (3)
Anticholinergics	1 (3)	0 (0)	1 (3)
GLP-1 agonists	0 (0)	0 (0)	1 (3)
Study for delayed GE ^a^			
Gastric scintigraphy	8 (24)	0 (0)	4 (10)
UGIS	0 (0)	0 (0)	5 (13)
Presenting symptom			
Nausea	33 (100)	2 (50)	35 (90)
Vomiting	31 (94)	3 (75)	30 (77)
Abdominal pain	9 (27)	1 (25)	11 (28)
Early satiety	8 (24)	1 (25)	7 (18)
Weight loss	6 (18)	0 (0)	6 (15)
Constipation	5 (15)	0 (0)	4 (10)
High residuals on NGT	0 (0)	0 (0)	2 (5)
No. of hospitalizations for gastroparesis or symptoms related to delayed GE			
0	20 (61)	3 (75)	31 (80)
1	12 (36)	1 (25)	8 (21)
2	1 (3)	0 (0)	0 (0)
4	1 (3)	0 (0)	0 (0)
Median time from ICI initiation to symptoms of delayed GE (IQR), months	--	10.2 (0.7–28.6)	12.8 (4.4–35.5)
Symptomatic treatment for presumed delayed GE, ^b^			
Domperidone	2 (6)	0 (0)	0 (0)
Metoclopramide	28 (85)	4 (100)	35 (90)
Macrolide	1 (3)	0 (0)	1 (3)
Steroids	1 (3)	0 (0)	0 (0)
Pyloric botulin	2 (6)	0 (0)	0 (0)
Transpyloric stenting	2 (6)	0 (0)	0 (0)
Supportive care			
NGT decompression	2 (6)	0 (0)	0 (0)
Parenteral nutrition	0 (0)	0 (0)	2 (5)
GJ or J tube	3 (9)	0 (0)	7 (18)
Clinical response/remission	9 (27)	1 (25)	14 (36)
Median duration of clinical symptoms of patients with clinical response/remission (IQR), days	--	2	108 (14.5–780)
Resumption of ICI therapy after symptoms of delayed GE	--	0 (0)	19 (49)

Note: All data are no. of patients (%) unless otherwise indicated. Abbreviations: Immune checkpoint inhibitor (ICI), gastric emptying (GE), esophagogastroduodenoscopy (EGD), gastrointestinal (GI), proton pump inhibitor (PPI), histamine-2 receptor antagonist (H2RA), glucagon-like peptide 1 (GLP-1), upper gastrointestinal series (UGIS), nasogastric tube (NGT), gastrojejunostomy (GJ), jejunostomy (J), interquartile range (IQR). ^a^ For patients with pre-existing gastroparesis without GES in our records, diagnosis was confirmed based on chart review and reconciliation of past medical history. ^b^ In the patients with pre-existing gastroparesis, all interventions preceded ICI therapy.

**Table 3:** Clinical disease course of patients with suspected delayed GE after immune checkpoint inhibitor (ICI) therapy based on presence of alternative etiology ^a^.

Characteristic	No Alternative Etiology, *n* = 12	Alternative Etiology, *n* = 27	*p*
Study for delayed GE			
Gastric scintigraphy	1 (8)	3 (11)	1.00
UGIS	0 (0)	5 (19)	0.299
Presenting symptom			
Nausea	9 (75)	26 (96)	0.078
Vomiting	7 (58)	23 (85)	0.102
Abdominal pain	5 (42)	6 (22.2)	0.262
Early satiety	4 (33)	3 (11)	0.172
Weight loss	2 (17)	4 (15)	1.00
Constipation	2 (17)	2 (7)	0.573
High residuals on NGT suctioning	0 (0)	2 (7)	1.00
Median time from ICI initiation to new onset of delayed GE (IQR), months	14.4 (6.1–26.3)	10.7 (1.4–39.3)	0.642
No. of hospitalizations for delayed GE–related symptoms			0.394
0	11 (92)	20 (74)	
1	1 (8)	7 (26)	
Gastroparesis medication			
Metoclopramide	11 (92)	24 (89)	0.640
Macrolide	0 (0)	1 (4)	--
Supportive care			0.417
Parenteral nutrition	1 (8)	1 (4)	
GJ or J tube	1 (8)	6 (22)	
HbA1c level (in diabetic patients)			1.00
<6.5%	2 (17)	4 (15)	
≥6.5%	3 (25)	9 (33)	
Clinical response/remission achieved	3 (25)	11 (41)	0.477
Median duration of clinical symptoms for patients with clinical response/remission (IQR), days	290 (147–387)	74.5 (21.5–690.0)	1.00
Resumption of ICI therapy after symptoms of delayed GE	5 (42)	14 (52)	0.731

Note: All data are no. of patients (%) unless otherwise indicated. Abbreviations: Gastric emptying (GE), upper gastrointestinal series (UGIS), nasogastric tube (NGT), immune checkpoint inhibitor (ICI), interquartile range (IQR), gastrojejunostomy (GJ), jejunostomy (J), hemoglobin A1c (HbA1c). ^a^ Alternative etiologies were diabetes, opioid use, glucagon-like peptide 1 receptor agonist use, prior bariatric procedures, and gastrointestinal malignancy.

## Data Availability

The raw data supporting the conclusions of this article will be made available by the authors upon request.

## Supplementary Materials

The following supporting information can be downloaded at: https://www.mdpi.com/article/10.3390/cancers16152658/s1, Table S1: Comparison of disease course between patients with suspected delayed gastric emptying (GE) after immune checkpoint inhibitor (ICI) therapy without an alternative etiology and patients with pre-existing gastroparesis without flare-up after ICI therapy, Figure S1: Gastric emptying study from post-ICI gastroparesis patient.
