# Supplementary material for: The Effect of Immune Checkpoint Inhibitor Therapy on Pre-Existing Gastroparesis and New Onset of Symptoms of Delayed Gastric Emptying
Source: Cancers (Basel). 2024 Jul 26;16(15):2658. doi: 10.3390/cancers16152658 (PMC11311627; doi:10.3390/cancers16152658)
Supplement: Supplementary file 1 [file cancers-16-02658-s001.zip › supplementary table S1.pdf]

Supplemental Table S1. Comparison of disease course between patients with suspected delayed gastric emptying (GE) after immune checkpoint inhibitor (ICI) therapy without an alternative etiology[a] and patients with pre-existing gastroparesis without flare-up after ICI therapy

Characteristic	Pre-existing gastroparesis without flare-up after ICI, n=33	Suspected delayed GE after ICI without an alternative etiology, n=12	*P*
Diagnostic study for delayed GE			
Gastric scintigraphy	8 (24)	1 (8)	0.407
UGIS	0 (0)	0 (0)	--
Presenting symptom			
Nausea	33 (100)	9 (75)	0.016
Vomiting	31 (94)	7 (58)	0.010
Abdominal pain	9 (27)	5 (42)	0.470
Early satiety	8 (24)	4 (33)	0.705
Weight loss	6 (18)	2 (17)	1.00
Constipation	5 (15)	2 (17)	1.00
High residuals on NGT	0 (0)	0 (0)	---
No. of hospitalizations for gastroparesis			0.123
0	20 (61)	11 (92)	
1	12 (36)	1 (8)	
2	0 (0)	0 (0)	
4	1 (3)	0 (0)	

Gastroparesis medication			1.00
Domperidone	2 (6)	0 (0)	
Metoclopramide	28 (85)	11 (92)	
Macrolide	1 (3)	0 (0)	
Steroids	1 (3)	0 (0)	
Intervention for delayed GE			--
Pyloric botulin	2 (6)	0 (0)	
Transpyloric stenting	2 (6)	0 (0)	
HbA1c level (in diabetic patients)			1.00
<6.5%	6 (18)	2 (17)	--
≥6.5%	7 (21)	3 (25)	--
Clinical response/remission	9 (27)	3 (25)	1.00

Note: All data are no. of patients (%) unless otherwise indicated.

Abbreviations: Gastric emptying (GE), upper gastrointestinal series (UGIS), nasogastric tube (NGT), hemoglobin A1c (HbA1c)

[a]Alternative etiologies were diabetes, opioid use, glucagon-like peptide 1 receptor agonist use, prior bariatric procedures and gastrointestinal malignancy.
