# Supplementary figures and images for: The Effect of Immune Checkpoint Inhibitor Therapy on Pre-Existing Gastroparesis and New Onset of Symptoms of Delayed Gastric Emptying
Source: Cancers (Basel). 2024 Jul 26;16(15):2658. doi: 10.3390/cancers16152658 (PMC11311627; doi:10.3390/cancers16152658)

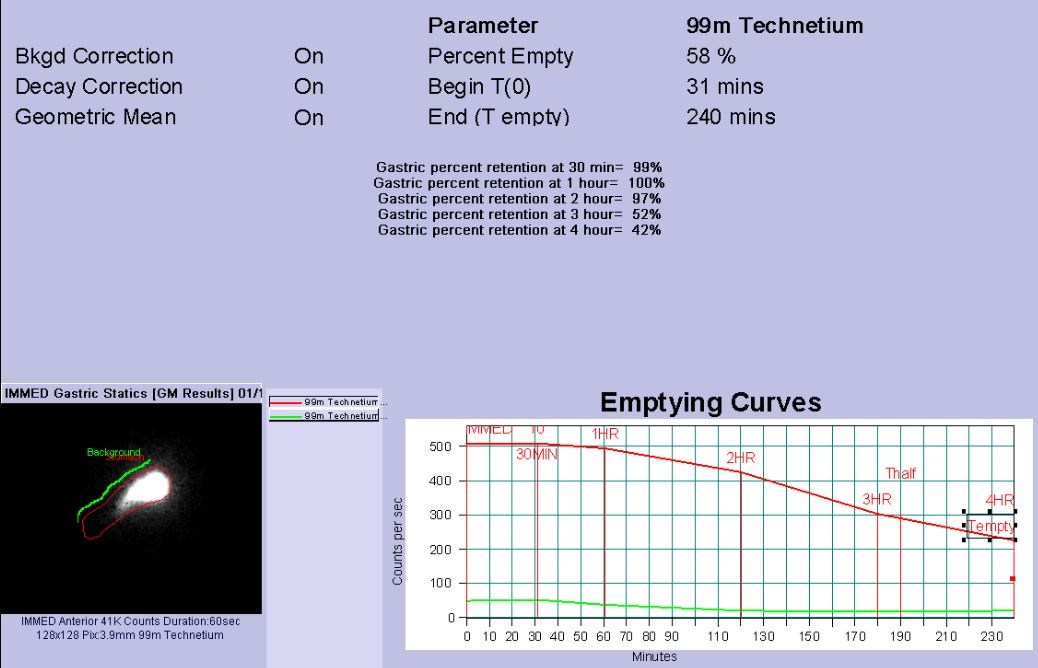

Supplement: Supplementary file 1 [file cancers-16-02658-s001.zip › Supplementary figure S1.JPG]
